# Perforation gastrique néonatale spontanée: à propos d’un cas

**DOI:** 10.11604/pamj.2018.30.72.13205

**Published:** 2018-05-29

**Authors:** Dimitri Kanyanda Nafatalewa, Jeff Bukasa Misenga, Eric Mbuya Musapudi, Pascal Monga Yebalaya, Didier Tshibangu Mujinga, Guy Nday Ilunga

**Affiliations:** 1 Université de Lubumbashi, Département de Chirurgie, Faculté de Médecine, Cliniques Universitaires de Lubumbashi, Service de Chirurgie, République Démocratique du Congo

**Keywords:** Perforation gastrique, nouveau-né, pneumopéritoine, Gastric perforation, newborn, pneumoperitoneum

## Abstract

La perforation gastrique néonatale spontanée est rare. Nous rapportons un cas survenu chez un nouveau-né de 4 jours issu d'une grossesse à terme. L’enfant a nécessité une réanimation à sa naissance. Au deuxième jour de sa vie, une distension abdominale importante était notée. La radiographie de l'abdomen sans préparation a montré un pneumopéritoine. La laparotomie a objectivé une perforation au niveau de la petite courbure gastrique de 1,5cm de diamètre qui a bénéficié d’une suture simple chirurgicale. L'évolution était marquée par le décès au premier jour post opératoire.

## Abstract (en)

Spontaneous neonatal gastric perforation is rare. We report the case of a full term 4-day old newborn who required resuscitation at birth. On the second day of life he had extensive abdominal distension. Abdominal x-ray without preparation showed pneumoperitoneum. Laparotomy showed a perforation at the level of the small gastric curvature, measuring 1,5 cm in diameter, treated by simple surgical suture. The patient dead on the first post-operative day.

## Introduction

La perforation gastrique spontanée est rare chez le nouveau-né à terme et représente 10 à 16% des perforations gastro-intestinales néonatales [1]. C’est une affection au pronostic grave. Le taux élevé de mortalité chez ces patients peut être amélioré par un diagnostic précoce et de réanimation rapide [2]. L'âge habituel de survenue de la perforation gastrique néonatale se situe entre deux et sept jours et il existe une prédilection pour la race noire et le sexe masculin [3]. Par ailleurs, plusieurs facteurs de risque sont associés à l'affection ; la prématurité, le faible poids de naissance, l'exsanguino-transfusion, la rupture prématurée des membranes, la toxémie gravidique, l'accouchement par le siège, le diabète maternel, le placenta prævia, l'infection amniotique ou encore la césarienne [2,4]. Dans la majorité des cas, elle nécessite une prise en charge chirurgicale précoce, pouvant aller de la suture chirurgicale à la gastrectomie partielle ou totale. Depuis la première description de Siebold en 1825 [3], plus de 300 cas ont été rapportés dans la littérature [4]. La rareté de cette pathologie dans le monde motive cette publication. L’objectif était de décrire le tableau clinique et la prise en charge d’un cas de perforation gastrique observé aux cliniques universitaires de Lubumbashi en Avril 2017.

## Patient et observation

Il s’agissait d’un nouveau-né de sexe masculin de 4 jours de vie, qui a été amené en consultation dans le service de chirurgie des cliniques universitaires de Lubumbashi en date du 20 avril 2017 pour ballonnement abdominal, pleurs incessant et fièvre. Dans les antécédents, il était né à terme dans un centre hospitalier de la place avec un score d’APGAR de 4 à la première minute et il a été réanimé pendant environ dix minutes. Il a présenté un ballonnement abdominal, fièvre et pleurs incessant depuis le premier jour de vie malgré l’émission du méconium. Il a été soumis sous céfotaxime, ampicilline, et le paracétamol. La persistance du ballonnement abdominal sans trouble du transit a motivé son transfert aux cliniques universitaires de Lubumbashi.

**Figure 1:**
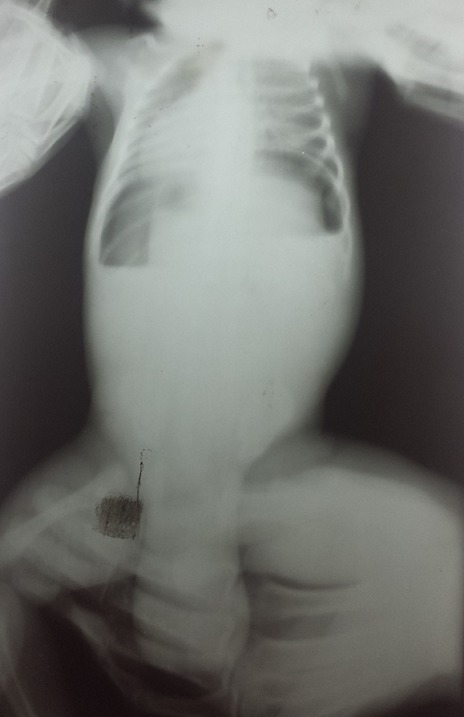
Radiographie abdomen sans préparation qui montre un large pneumopéritoine

A l’examen physique fait aux cliniques universitaires de Lubumbashi, les paramètres anthropométriques se présentaient comme suite: poids: 3950 gr, périmètre crânien: 36cm, périmètre thoracique: 36cm, périmètre brachial: 12cm et la taille: 53cm. L’examen somatique avait montré une pâleur tégumentaire avec un temps de recoloration cutanée inférieur à 3 secondes, un abdomen ballonné, une défense abdominale, avec sensibilité diffuse à la palpation, un tympanisme, le péristaltisme était présent et il n’y avait pas d’organomegalie. L’examen des organes génitaux externes avait objectivé une tuméfaction de l’hemibourse gauche, le testicule non palpé, le test à la transllumination était positif. Devant ce tableau une radiographie abdominale sans préparation avait été réalisée et avait révélé un pneumopéritoine (Figure 1). L’échographie avait montré un foie de volume normal, décollé du diaphragme par une collection hypoechogène avec aspect de granule hypoechogène; la cavité abdominale remplie d’une collection hypoechogène, plus abondant en sous mesocolique et comprimant les anses visibles en profondeur et non dilatés. La même collection était visible dans les 2 hemibourses mais plus abondante à droite. Les testicules étaient visibles dans les 2 hemibourses et de volume normal.

**Figure 2:**
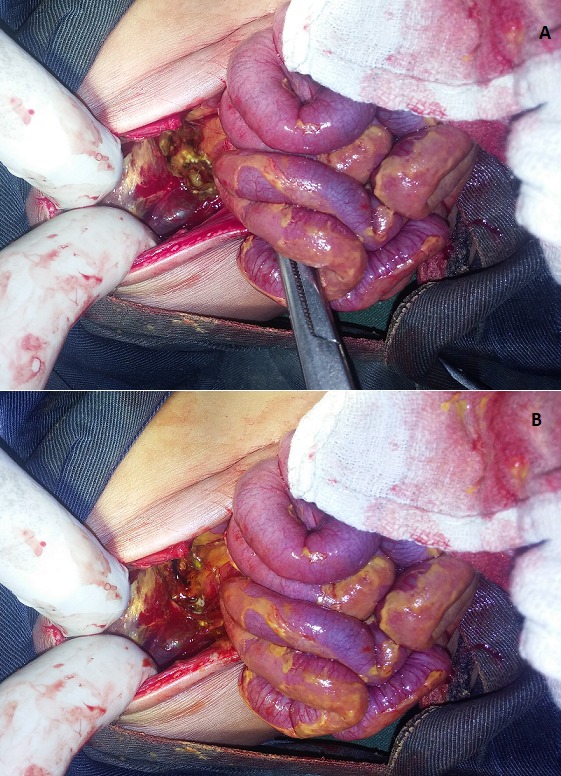
(A, B) exposition per opératoire de la perforation gastrique sur la petite courbure

Les diagnostics d’une péritonite, d’une hydrocèle gauche communicante, et d’infection néonatale précoce étaient retenus et une laparotomie exploratrice avait été décidée. Une prise de sang a montré un groupe sanguin B rhésus positif, hémoglobine à 17g%, l’hématocrite à 58%. Après une visite pré-anesthésique et une réanimation préopératoire, il a été opéré un jour après son admission. A l’ouverture de la cavité abdominale, une issu d’un liquide d’aspect verdâtre, d’odeur aigrelette était mis en évidence. La présence d’adhérence papyracée en sus mesocolique, a justifié l’adhésiolyse par digitoclasie. Le relèvement du bord antérieur du foie, a permis de mettre en évidence une brèche ovalaire d’environ 1,5cm de diamètre au niveau de la petite courbure de l’estomac (Figure 2A et Figure 2B). L’aspiration du liquide, l’adhesiolyse, la suture en bourse de la brèche suivie d’une plicature serosereuse (suture de décharge) au vicryl N°2/0, le nettoyage abondant de la cavité abdominale au sérum physiologique et un drainage de l’étage sus-mesocolique et du Douglas avaient été réalisés. En post-opératoire, le nouveau-né a été pris en charge par une équipe mixte associant, pédiatres, réanimateurs et chirurgiens. Il avait bénéficié d’une antibiothérapie fait de ceftriaxone et gentamycine, une transfusion, un apport liquidien en perfusion, une analgésie et un réchauffement à la bouillotte. Il était décédé un jour après l’opération soit au 6^e^ jour de vie dans un tableau de choc avec une détresse respiratoire.

La perforation gastrique néonatale idiopathique ou primitive est connue depuis la première observation rapportée par Siebold en 1825. Elle représente 10 à 16% des perforations gastro-intestinales néonatales [3]. Sa mortalité est lourde [1]. L'âge habituel de survenue se situe entre deux et sept jours et il existe une prédilection pour la race noire et le sexe masculin [1]. Abdelhalim Naji et all (2014) au Maroc rapporte un cas d’un nouveau-né de 2 jours [4]; Giovanni DR et all (2014) en Inde ont rapporté un cas [5]; Mustafa Aydin et all (2015) rapportent en Turquie un cas d’un prématuré de 2 jours [2]; Yong Hoon Cho et all (2015) en Corée a décrit une observation de 11 cas pendant 10 ans soit de janvier 2005 à décembre 2014, dont l’âge gestationnel moyen était de 29.7 ± 3.7 semaines d’aménorrhée [6].

Notre patient était un nouveau-né de 3 jours de vie, de sexe masculin et de race noire. Plusieurs causes sont avancées dans la survenue de la perforation. Ainsi, ont été rapportées les perforations d'origine congénitale par agénésie de la musculature gastrique occasionnant des lésions à type de déchirure linéaire au niveau de la grande courbure; puis les perforations d'origine ischémique (souffrance néonatale, embole septique), d'origine mécanique (distension gastrique après ventilation au masque trop appuyée, sonde gastrique perforant l'estomac), d'origine médicamenteuse (corticoïdes, indométacine dans la communication interauriculaire) ou encore d'origine fonctionnelle (affection neurologique, atonie gastrique, spasme pylorique en cas de stress néonatal) entraînant des perforations punctiformes de la paroi gastrique antérieure ou postérieure. Par ailleurs, plusieurs facteurs de risque sont associés à l'affection: la prématurité, le faible poids de naissance, l'exsanguino-transfusion, la rupture prématurée des membranes, la toxémie gravidique, l'accouchement par le siège, le diabète maternel, le placenta prævia, l'infection amniotique ou encore la césarienne [6-8]. Parmi les facteurs étiologiques répertoriés dans la littérature, les facteurs retrouvés dans notre observation étaient: un score APGAR déprimé, une souffrance néonatale, une réanimation.

Dans cette pathologie on décrit deux modes de présentation. Le premier est un tableau de pneumopéritoine qui est souvent associé à un tableau de choc. Ce tableau oblige un traitement chirurgical et son pronostic est péjoratif; Le deuxième, un tableau d’hémorragie digestive massive pour lequel le traitement médical peut être suffisant [9]. Dans notre cas, il s’agissait d’un tableau de pneumopéritoine car la radiographie de l’abdomen sans préparation avait mis en évidence un pneumopéritoine. En outre, nous n’avons pas objectivé d’hémorragie digestive. Sur le plan thérapeutique, nous avons recouru en une suture en bourse, un drainage de l’étage sus mesocolique ainsi que du Douglas avaient été effectués. Ce traitement est décrit par plusieurs auteurs [6, 8]. Pour les cas diagnostiqués et traités précocement l’évolution est favorable. Cependant l’évolution est défavorable pour les cas pris en charge tardivement [3]. Pour notre cas, l’évolution s’est soldée par un décès au premier jour post opératoire suite au retard de prise en charge et à une défaillance multiviscerale.

## Conclusion

La perforation gastrique néonatale est une entité rare. Les praticiens devront y penser en vue d’une prise en charge précoce dans le but d’améliorer le pronostic.

## Conflits d’intérêts

Les auteurs ne déclarent aucun conflit d'intérêt.

## References

[1] Rakoto-Ratsimba HN, Rakotoarisoa B, Samison LH, Belalahy V, Ranaivozanany A (2004). Spontaneous gastric perforation in a neonate: a case report. Arch Pediatr.

[2] Mustafa Aydin (2011). Gastric perforation in an extremely low birth weight infant recovered with percutaneous peritoneal drainage. The Turkish Journal of Pediatrics.

[3] Abdelhalim Naji, Coll (2015). Perforation gastrique néonatale spontanée: à propos d’un cas. Pan Afr Med J.

[4] Gharehbahgy MM, Rafeey M (2001). Acute gastric perforation in neonatal period. Med J Islamic Academy Sci.

[5] Govani DR (2014). Pneumo-omentocele - a Sign of Silent Lethal Neonatal Posterior Gastric Perforation. Austin J Clin Case Rep.

[6] Terui K, Iwai J, Yamada S, Takenouchi A, Nakata M, Komatsu S, Yoshida H (2012). Etiology of neonatal gastric perforation: a review of 20 years' experience. Pediatr Surg Int.

[7] Yong Hoon Cho (2015). Gastric Perforation in the Neonatal Period: Differences between Preterm and Term Infants. Original Article Néonatal Med.

[8] Kuremu RT, MBCHB et Coll (2004). Neonatal gastric perforation. East African Medical Journal. January.

[9] Mallet EC et Coll (1999). Perforation gastrique néonatale spontanée. Service de néonatologie et réanimation pédiatrique.

